# The Agreement of Readability and Interpretability of Fetal Heart Rate Between Mobile and Conventional Cardio-Tocography

**DOI:** 10.3390/diagnostics16121803

**Published:** 2026-06-11

**Authors:** Pajaree Ruenpeng, Suchaya Luewan, Threebhorn Kamlungkuea, Masaaki Tokuda, Kazuhiro Hara, Kenji Kanenishi, Theera Tongsong

**Affiliations:** 1 Department of Obstetrics and Gynecology, Faculty of Medicine, Chiang Mai University, Chiang Mai 50200, Thailand; pajaree.ru@gmail.com (P.R.); threebhorn.k@cmu.ac.th (T.K.); theera.t@cmu.ac.th (T.T.); 2 Fetal Center, Faculty of Medicine, Chiang Mai University, Chiang Mai 50200, Thailand; 3 NPO Electronic Health Care Innovation in Kagawa, Takamatsu 760-0023, Japan; tokuda.masaaki@kagawa-u.ac.jp (M.T.); hara.kazuhiro@kagawa-u.ac.jp (K.H.); 4 Faculty of Medicine, Kagawa University, Kagawa 761-0793, Japan; kanenishi.kenji@kagawa-u.ac.jp

**Keywords:** cardiotocography, conventional cardiotocography, fetal heart rate, mobile cardiotocography, nonstress test

## Abstract

**Background/Objective:** Conventional cardiotocography (CTG) is an essential tool for fetal surveillance but remains inaccessible in many low-resource settings. Emerging low-cost, wireless mobile CTG systems designed for home-based use may enhance global access to fetal monitoring. Nevertheless, their concordance with conventional CTG in the interpretation of FHR patterns remains insufficiently validated. This study aimed to evaluate agreement in the readability and interpretability of FHR baseline patterns and accelerations/decelerations between mobile and conventional CTG. **Methods**: A prospective study was conducted in pregnant women undergoing antepartum surveillance to evaluate agreement in the interpretation of FHR tracings simultaneously obtained from mobile and conventional CTG. Each paired tracing was independently and blindly assessed by two physician reviewer groups. **Results:** A total of 404 women underwent simultaneous assessment with both modalities. Agreement in baseline FHR interpretation was excellent between reviewer groups and modalities (ICC > 0.95). Interpretation of accelerations and decelerations also demonstrated very good agreement (κ = 0.8–0.9), whereas agreement for FHR variability and uterine contractions was moderate. Both reviewer groups assigned slightly but significantly higher quality scores and satisfaction scores to conventional CTG compared with mobile CTG. **Conclusions**: Mobile CTG demonstrates excellent agreement with conventional CTG in the assessment of FHR baseline and accelerations/decelerations. However, the overall quality and user satisfaction associated with mobile CTG were slightly lower than those observed with conventional CTG. Because of its advantages, including lower cost, portability, and user-friendly design, mobile CTG may serve as a feasible alternative for antepartum surveillance in low-resource settings worldwide.

## 1. Introduction

Stillbirth remains a major global health burden, with an estimated 1.9 million cases in 2021, equivalent to one death every 16 s [1]. Key contributing factors include maternal comorbidities, such as diabetes, and hypertension, as well as limited access to quality antenatal care, particularly in rural settings. Antenatal fetal heart rate (FHR) testing, or non-stress testing (NST), has been widely used for more than five decades to assess fetal well-being by identifying heart rate patterns associated with fetal hypoxia and autonomic dysfunction [2,3,4,5]. A normal NST is highly reassuring, with a negative predictive value of 99.8% and a stillbirth rate of 1.9 per 1000 within one week [6,7]. Given its non-invasive nature and clinical utility, NST is extensively employed, especially in high-risk pregnancies [6,8].

FHR monitoring has evolved from intermittent auscultation to continuous cardiotocography (CTG) [9,10,11]. CTG records FHR and uterine activity via external abdominal sensors, while NST specifically evaluates FHR response to fetal movement without inducing contractions. According to NIH criteria, NST results are classified as reactive or non-reactive. A reactive NST (≥32 weeks) requires at least two accelerations (≥15 bpm for ≥15 s within 20 min), indicating adequate oxygenation, whereas a non-reactive result necessitates further evaluation. FHR variability, an essential parameter reflecting autonomic function, is categorized as absent, minimal (≤5 bpm), moderate (6–25 bpm; normal), or marked (>25 bpm) [12,13,14].

Recent advances in wireless technology have facilitated the development of mobile FHR monitoring systems. The HeraBEAT device has demonstrated acceptable accuracy and usability; however, its performance is not equivalent to that of NST [15]. The Pregnabit system has shown feasibility and diagnostic concordance with conventional CTG in a Polish cohort [16]. Similarly, the iCTG device demonstrated feasibility and high user satisfaction, although comparative evaluation was not performed [17]. Moreover, mobile CTG has been reported to provide reliable antepartum and intrapartum monitoring, with the potential to enhance access to fetal surveillance in peripheral settings and facilitate earlier identification of high-risk pregnancies [18].

Mobile CTG represents a promising modality for remote, home-based, and transfer settings; however, evidence regarding its agreement with conventional CTG remains limited. Although prior studies have emphasized its accessibility and portability, direct comparisons of clinical interpretability with conventional CTG are scarce. We hypothesized that mobile CTG would exhibit a high level of agreement with conventional CTG in fetal heart rate (FHR) interpretation. Accordingly, the primary objective was to assess agreement in the readability and interpretability of FHR baseline and accelerations/decelerations between the two modalities. Secondary objectives included evaluating agreement in FHR variability, as well as comparing tracing quality and interpreter satisfaction.

## 2. Patients and Methods

**Design and population:** A single-center, prospective cross-sectional comparative study was conducted at the antenatal care clinic, Maharaj Nakorn Chiang Mai Hospital, a tertiary referral and teaching hospital, Chiang Mai University, Thailand, between August 2025 and April 2026. The study population comprised pregnancies requiring antenatal surveillance due to maternal and/or fetal indications. Ethical approval was obtained from the Institutional Review Board of Chiang Mai University (Research Ethics Committee Panel 5; OBG-2568-0467; Research ID: 0467; approved on 29 July 2025). The inclusion criteria were as follows: (1) singleton pregnancy; (2) maternal age ≥ 20 years; (3) gestational age between 28 and 40 weeks; and (4) indications for antenatal surveillance based on maternal and/or fetal conditions, in accordance with the American College of Obstetricians and Gynecologists (ACOG) recommendations [6,8]. The exclusion criteria were as follows: (1) preterm premature rupture of membranes; (2) pre-pregnancy body mass index (BMI) ≥35 kg/m^2^ or ≤15 kg/m^2^; (3) fetal structural or chromosomal abnormalities; (4) presence of an implanted electronic device (e.g., pacemaker); (5) emergency obstetric conditions or the need for urgent treatment, such as active antepartum hemorrhage; and (6) withdrawal of consent to participate in the study.

**Data Collection**: Pregnant women who met the inclusion criteria were invited to participate and provided written informed consent. During the procedure, participants were positioned in a semi-recumbent or left lateral tilt position, while full supine positioning was avoided to maintain adequate placental perfusion. NST was performed simultaneously using conventional CTG and mobile CTG devices by well-trained physicians and the research team. The conventional CTG used to monitor FHR and uterine activity was the F9 Express cardiotocography system (Edan Instruments, Inc., Shenzhen, China), equipped with a 12-crystal 1.0 MHz Doppler transducer, dual FHR channels, TOCO monitoring, and integrated maternal vital-sign measurement, with data recorded via a built-in thermal printer and digital waveform storage. The mobile CTG was a mobile cardiotocography system (iCTG, Melody International Ltd., Takamatsu, Japan), consisting of wireless Doppler and TOCO transducers connected via Bluetooth to a tablet device, enabling real-time fetal heart rate and uterine activity monitoring with cloud-based remote data transmission. For external monitoring, a Doppler ultrasound transducer was placed over the area of maximal fetal heart sound intensity, typically along the fetal back, with conductive gel applied to optimize signal acquisition. A tocodynamometer was positioned over the uterine fundus to monitor uterine contractions. Both transducers were secured with elastic belts, and FHR and uterine activity were recorded for approximately 20–30 min. Throughout the electronic fetal heart rate monitoring period, a research nurse provided guidance, assisted with device application, and continuously supervised the monitoring process. The device was checked regularly and repositioned or reapplied when necessary to ensure proper placement and optimal signal acquisition. Clinical management followed standard CTG interpretation protocols. For study purposes, CTG tracings from both devices were independently reviewed by the research team after completion of data collection. All tracings were analyzed in a randomized manner by the two groups of researchers to minimize interpretation bias, with each group comprising three independent researchers. Both groups assessed both the mobile and conventional CTG recordings. Collected data included clinical characteristics, such as age, parity, gestational age and maternal weight at the time of NST, educational level, pre-pregnancy BMI, and indications for antenatal testing. CTG parameters included baseline FHR, short-term variability (absent, minimal, moderate, or marked), accelerations (the presence of at least 2 times), decelerations, uterine contractions, and overall satisfaction assessed using a 5-point Likert scale (very satisfied = 5, satisfied = 4, neutral = 3, dissatisfied = 2, and un-interpretable = 1).

**Outcome Measures:** The primary outcome was the level of agreement between the two modalities regarding the readability and interpretability of baseline FHR and FHR accelerations/decelerations. Secondary outcomes included agreement in FHR variability, as well as comparisons of tracing quality and interpreter satisfaction. Tracing quality was categorized as poor, acceptable, and good.

**Statistical Analysis:** The estimated sample size was calculated based on the method described by Donner et al. [19]. To achieve a power of 90% at a two-sided significance level (α-error) of 0.05, assuming an expected high level of agreement with a Kappa coefficient of 0.90 and a minimum acceptable Kappa coefficient of 0.75, a minimum sample size of 410 pairs of FHR tracings (205 cases) was required, including an anticipated 10% proportion of non-reactive tracings.

Baseline data were summarized as mean ± SD for continuous variables and as proportions for categorical variables. Regarding NST readability, baseline FHR and uterine contraction parameters, including duration and interval, were presented as mean ± SD, whereas variability categories (absent, minimal, moderate, and marked), accelerations, decelerations, and contraction consistency were presented as proportions. NST interpretability (reactive or non-reactive) was also expressed as proportions. Tracing quality and overall satisfaction, assessed using a Likert scale, was presented as median with interquartile range (IQR). For comparative analyses, continuous variables were analyzed using the paired Student’s *t*-test or Wilcoxon signed-rank test, as appropriate according to data distribution, while categorical variables were compared using McNemar’s chi-square test. Inter-rater agreement was evaluated using Cohen’s kappa statistic; and the intraclass correlation coefficient (ICC) based on a two-way random-effects model with an absolute agreement. A *p*-value < 0.05 was considered statistically significant. Statistical analyses were performed using Stata (Version 16.0, StataCorp LLC, College Station, TX, USA) and IBM SPSS Statistics (Version 26.0, IBM Corp., Armonk, NY, USA).

## 3. Results

During the study period, a total of 404 women were included, generating 404 paired CTG recordings (808 tracings in total). The mean maternal age was 31.3 ± 6.0 years, and most participants were nulliparous (67.3%). The mean gestational age at NST was 35.1 ± 3.1 weeks. The mean pre-pregnancy BMI was 24.6 ± 6.4 kg/m^2^, while the mean maternal weight at the time of NST was 63.0 ± 17.5 kg. Regarding educational attainment, approximately 60% of participants had a bachelor’s degree or higher. The most common indications for antenatal surveillance were fetal growth restriction (FGR; 25.7%) and gestational diabetes mellitus (23.3%), followed by non-reassuring fetal status (11.6%), and hypertensive disorders (8.4%). Of them, there were non-reactive NST (no acceleration) 32 cases, and 6 cases had abnormal FHR variability (5 minimal and 1 absent). Detailed baseline characteristics are presented in Table 1.

**Figure 1:**
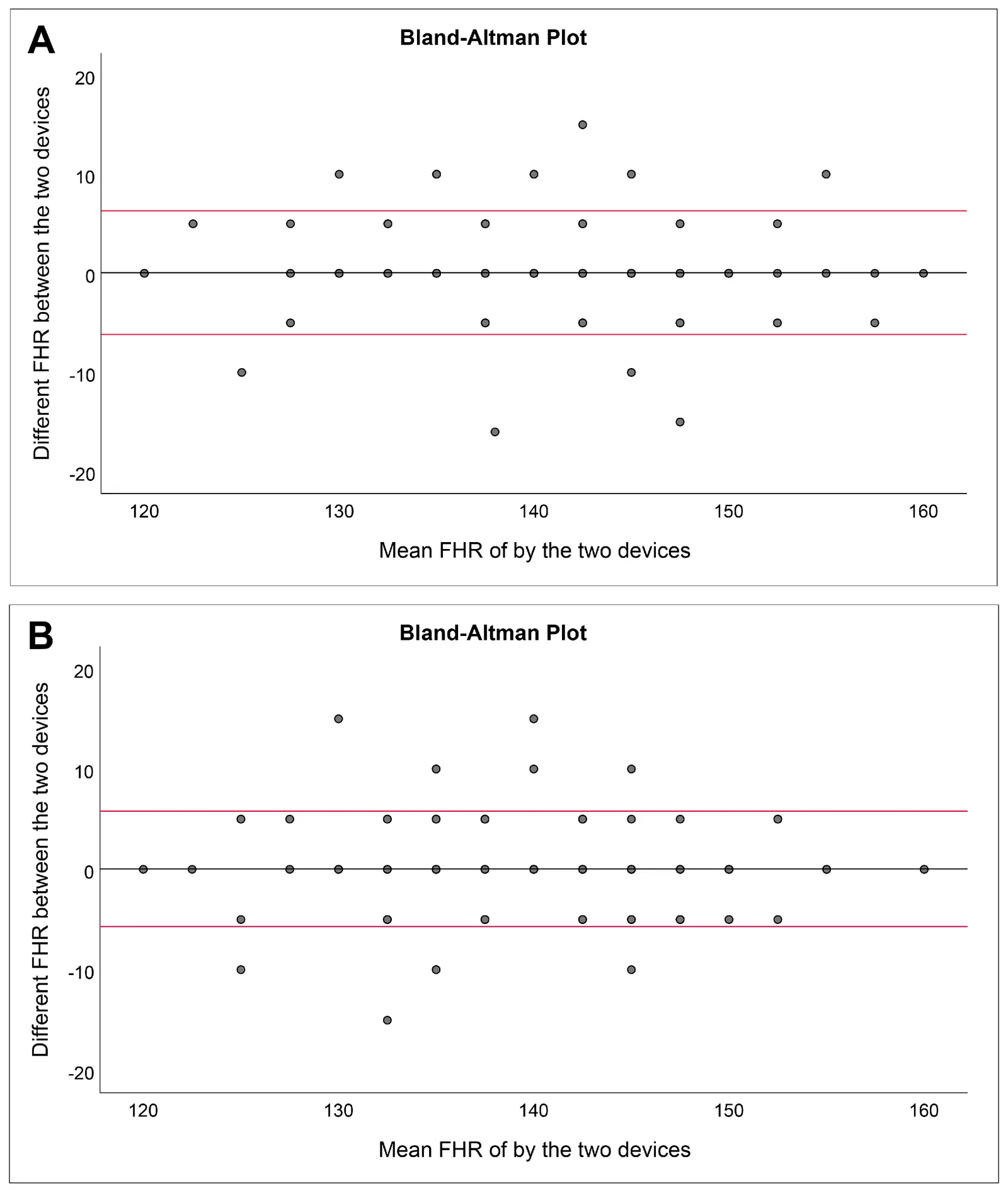
Bland–Altman analysis of inter-device reliability by interpreter group. Panel (**A**) represents Group 1 interpreters (mean difference 0.072 [95% CI: −0.24 to 0.38], SD: 3.18; *p*-value: 0.650), and panel (**B**) represents Group 2 interpreters (mean difference 0.037 [95% CI: −0.25 to 0.38], SD: 2.94; *p*-value: 0.800).

**Inter-rater agreement:** For the assessment of agreement between reviewer groups in the interpretation of baseline FHR, the ICC demonstrated excellent reliability for both modalities, with ICC values of approximately 0.95 for both mobile and conventional CTG. Detailed results are presented in Table 2. Bland–Altman analysis of inter-device reliability stratified by interpreter group demonstrated good agreement between the two devices. The mean difference in FHR measurements was not significantly different from zero in either group, indicating the absence of systematic bias and clinical acceptance (Figure 1). For categorical data, inter-rater agreement was evaluated between two independent reviewer groups for each modality, with separate assessments performed for mobile CTG tracings (MR1 vs. MR2) and conventional CTG tracings (CR1 vs. CR2). Overall, both the percentage agreement and kappa coefficients demonstrated excellent agreement between reviewers in the interpretation of FHR parameters for both modalities. However, although interpretation of FHR variability showed very high percentage agreement between reviewer groups for both mobile and conventional CTG (99.80% for both modalities), the corresponding kappa coefficients were relatively lower (0.555 and 0.692, respectively) as presented in Table 2. This discrepancy was likely attributable to the low prevalence of abnormal variability patterns, particularly absent or minimal variability.

**Inter-modality agreement:** For the assessment of agreement between the two modalities in the interpretation of baseline FHR by the independent two reviewer groups, the intraclass correlation coefficient (ICC) demonstrated excellent reliability, with ICC values exceeding 0.90 across all reviewer groups. Detailed results are presented in Table 3.

For categorical variables, inter-modality agreement between mobile and conventional CTG was assessed by two independent reviewer groups. High percentages of agreement were observed across all parameters and were comparable between the reviewer groups. However, relatively lower kappa coefficients were noted for the interpretation of FHR variability and uterine contractions.

For uterine contraction detection, overall agreement between mobile and conventional CTG was high in both reviewer groups (90.9% and 92.3%, respectively); however, Cohen’s kappa was only moderate (0.53 and 0.60). Discordant cases occurred in both directions, some contractions were detected only by mobile CTG and others only by conventional CTG, indicating no consistent superiority of either modality.

Although percent agreement for FHR variability was high across both rater groups and modalities, Cohen’s kappa values were relatively low. This discrepancy was likely due to the low prevalence of abnormal variability (minimal and absent variability), resulting in imbalanced marginal distributions. Therefore, Gwet’s AC was used to assess agreement, and the results are presented in Table 4. The finding of high Gwet’s AC values despite relatively low kappa coefficients suggests that the raters demonstrated substantial agreement, and that the lower kappa values were likely an artifact of category imbalance rather than a true reflection of poor inter-rater reliability.

**Quality Assessment:** The quality of FHR tracings was independently assessed by two reviewer groups using a Likert scale (0 = poor, 1 = acceptable, and 2 = good). Both reviewer groups assigned slightly but significantly higher quality scores to conventional CTG compared with mobile CTG, as presented in Table 5.

**Satisfaction Assessment:** Satisfaction assessment was independently performed by two reviewer groups using a 5-point Likert scale (1 = very dissatisfied and 5 = very satisfied). Comparable satisfaction scores were observed between the two reviewer groups for both mobile and conventional CTG modalities. Nevertheless, both reviewer groups assigned slightly but significantly higher satisfaction scores to conventional CTG compared with mobile CTG, as presented in Table 6.

## 4. Discussion

The key insights derived from this study are as follows: (1) Agreement in FHR interpretation was excellent between mobile and conventional CTG, as well as among interpreters. (2) Agreement in the assessment of FHR variability between the two modalities was very good; however, conclusions regarding minimal and absent variability remain inconclusive due to the limited sample size within these subgroups. (3) Interpretation of FHR accelerations and decelerations demonstrated very good agreement between the two modalities. (4) Agreement for uterine contraction detection between mobile and conventional CTG was moderate. However, discordant results occurred in both directions, and neither modality consistently detected more contractions than the other. (5) Based on subjective assessment, the quality of FHR tracings obtained using mobile CTG was slightly but significantly lower than that of conventional CTG, although this difference is unlikely to be clinically meaningful. (6) Similarly, interpreter satisfaction with mobile CTG tracings was marginally but significantly lower than with conventional CTG, with no apparent clinical significance. However, although this difference reached statistical significance, it is unlikely to be clinically meaningful because the absolute difference in satisfaction scores was very small. The principal finding of this study is that mobile CTG demonstrated high agreement with conventional CTG in providing clinically meaningful information, particularly with respect to baseline FHR and accelerations/decelerations, key parameters that constitute the core components of antepartum surveillance (NST).

Although the kappa coefficients for accelerations and decelerations were in the very good range, occasional discrepancies between modalities or reviewers may still occur in clinical practice. From a practical perspective, the observed level of agreement suggests that mobile CTG can reliably support routine antepartum surveillance. However, clinical management should continue to incorporate the overall maternal and fetal condition rather than relying solely on CTG classification.

It is noteworthy that the inter-rater agreement for FHR variability, as measured by the kappa statistic, was relatively low for both mobile and conventional CTG, whereas agreement between the two modalities was comparatively higher. Substantial inter-rater disagreement was observed regardless of the device used, suggesting that the disagreement is not solely device-related. FHR tracing interpretation is inherently subjective, and disagreement remains common even when the same standard CTG device is used. In particular, distinguishing between absent and minimal variability is challenging and often results in lower agreement than the assessment of normal or marked variability, even among the experts [20,21]. Therefore, discrepancies in the interpretation of abnormal variability are more likely attributable to the subjective nature of FHR assessment and the characteristics of the tracings than to differences between devices.

The quality of FHR tracings and interpreter satisfaction may be marginally lower with mobile CTG than with conventional CTG; however, this difference is unlikely to be clinically meaningful given the high level of agreement between the two methods. One possible explanation is related to the physical characteristics of the monitoring devices. Compared with conventional CTG systems, which are generally larger, more firmly belt-fixed, and mechanically stable, mobile CTG devices are designed to be lightweight, compact, and highly portable. As a result, mobile CTG may be more susceptible to maternal movement, respiration, fetal movement, temporary signal dropout, motion-related noise, or minor variations in transducer contact during monitoring. Nevertheless, despite these potential technical limitations, agreement for clinically important FHR parameters remained consistently high between the two modalities. Concordance was particularly strong for key parameters, baseline FHR and accelerations/decelerations, which constitute the principal components of antenatal assessments (NST). Although this study demonstrates high agreement between mobile and conventional CTG for clinically important FHR interpretation, mobile CTG also offers several practical advantages over conventional systems, including portability, user-friendly operation, lower cost, digital connectivity, and home-based application. Consequently, broader availability of this modality may facilitate access to antenatal fetal surveillance in low-resource settings worldwide.

The relatively lower agreement for uterine contractions deserves careful interpretation. Although the kappa coefficient for uterine contraction detection was only moderate, the overall agreement between mobile and conventional CTG remained high. This apparent discrepancy is likely explained, at least in part, by the prevalence effect on kappa statistics. Because contraction-positive tracings were relatively infrequent, a large proportion of paired recordings were classified as negative by both modalities, thereby increasing crude percentage agreement while lowering the kappa coefficient after adjustment for chance agreement. Importantly, discordant cases were observed in both directions, with some contractions detected only by conventional CTG and others only by mobile CTG, suggesting no systematic bias toward either modality. In addition, conventional external tocodynamometry is not a true gold standard for uterine contraction detection, particularly during antepartum surveillance. Therefore, the observed discrepancies are more likely to reflect intrinsic inter-method variability than inferior performance of mobile CTG.

**The strengths** of this study are as follows. The findings were derived from real-world clinical practice and prospectively collected data. Although previous studies have shown satisfactory performance of mobile devices [15,16,17,18], our study differed in that the design allowed for precise paired comparisons, as tracings from both methods were recorded simultaneously under identical conditions. In particular, the report by Tamaru et al. using the same mobile CTG system demonstrated the feasibility of self-guided fetal heart rate monitoring with extremely high rates of readable recordings and high maternal satisfaction [17]. However, those studies mainly focused on usability and feasibility rather than direct comparison with conventional CTG. The present study extends these findings by demonstrating high agreement between mobile and conventional CTG in the interpretation of clinically important FHR parameters. Furthermore, inter-rater variability was assessed across a large and diverse group of physicians, enhancing the representativeness of routine practice. This approach strengthens the generalizability and reproducibility of the findings.

**The limitations** of this study should be acknowledged. First, although the sample size was adequate for the primary objective, it was insufficient for a robust assessment of agreement in certain FHR variability categories, particularly minimal and absent variability. Second, both monitoring modalities were applied simultaneously, unlike routine clinical practice. This may have compromised optimal transducer positioning, particularly for uterine contraction monitoring, where the optimal detection area is relatively limited. Consequently, contraction detection performance in both systems may have been underestimated compared with routine use, in which only one device would typically be applied and optimally positioned. Third, complete blinding to device type was not possible because the tracings generated by the two systems had distinct visual appearances, potentially introducing bias. Fourth, this study evaluated agreement in tracing interpretation rather than maternal or neonatal outcomes. Future studies should assess whether implementation of mobile CTG yields comparable clinical outcomes, improved access to fetal surveillance, and cost-effectiveness in real-world settings. Finally, women with marked obesity were excluded from this study. Therefore, the findings may not be generalizable to populations with class III obesity.

Nevertheless, the primary objective of this study was to determine whether mobile CTG differs from conventional CTG in its ability to detect and interpret FHR patterns, the core clinical function of cardiotocography. Our findings demonstrated no clinically meaningful differences between the two modalities in the assessment of baseline FHR or accelerations and decelerations. Given their comparable performance in essential FHR monitoring, the additional advantages of mobile CTG, including portability, battery-powered operation, lightweight design, digital data storage, internet-based data transmission, and the potential for future AI-assisted interpretation, may substantially broaden its applicability, particularly in remote, resource-limited, home-based, and telemedicine settings. Although the limited availability of experienced interpreters may remain a challenge in some locations, telemedicine could facilitate remote consultation with specialists at larger healthcare centers through digital communication networks.

**Clinical implications**: The findings of this study support the integration of mobile CTG into antenatal care as a viable alternative to conventional CTG for NST in the primary screening of fetal well-being, demonstrating high agreement in FHR baseline and periodic changes. Owing to its lower cost, greater accessibility, portability, home-based application, and user-friendly design, mobile CTG is particularly well suited for implementation in resource-limited settings. These findings may contribute to improved access to fetal surveillance among high-risk pregnancies in remote and underserved regions, with the potential to reduce adverse outcomes through earlier detection and timely intervention.

**Research implications**: Further studies are warranted to evaluate the level of agreement in the interpretation of FHR variability between mobile CTG and conventional CTG. Future studies employing only one device at a time may provide a more accurate assessment of uterine contraction detection by minimizing interference with optimal transducer placement. In addition, large-scale investigations are needed to assess the extent to which the implementation of mobile CTG may expand the coverage of antenatal fetal surveillance across geographically dispersed and resource-limited settings.

## 5. Conclusions

Mobile CTG demonstrates excellent agreement with conventional CTG in the assessment of FHR baseline and accelerations/decelerations. Because of its advantages, including lower cost, portability, home-based application, and user-friendly design, mobile CTG may serve as a feasible alternative for antepartum surveillance in low-resource settings worldwide. However, mobile CTG received slightly lower ratings for overall tracing quality and user satisfaction than conventional CTG, although these differences are unlikely to be clinically meaningful. In addition, agreement in the interpretation of FHR variability remained inconclusive and warrants further validation in future studies.

## Figures and Tables

**Table 1:** Baseline characteristics.

Variables	Total (*n* = 404)
Age (years), mean ± SD	31.3 ± 6.0
Parity, *n* (%)	
Nulliparous	272 (67.3%)
Multiparous	132 (32.7%)
Gestational age at NST (weeks), mean ± SD	35.1 ± 3.1
Pre-pregnancy BMI (kg/m^2^), mean ± SD	24.6 ± 6.4
Weight at NST (kg), mean ± SD	63.0 ± 17.5
Education level, *n* (%)	
Primary or less	22 (5.4%)
Secondary	139 (34.4%)
Bachelor or higher	243 (60.1%)
Indication for antenatal surveillance, *n* (%)	
Fetal growth restriction	104 (25.7%)
Hypertensive disorders	34 (8.4%)
Pregestational diabetes mellitus	15 (3.7%)
Gestational diabetes mellitus	94 (23.3%)
Non-reassuring fetal status	47 (11.6%)
Abnormal amniotic fluid volume	28 (5.9%)
Others	82 (20.3%)

**Table 2:** Inter-raters for interpretation of fetal heart rate tracings.

Continuous Variables *	**ICC**	**95% CI**	***p*-Value**
FHR (*MR1 vs. MR2*)	0.948	0.937–0.958	<0.001
FHR (*CR1 vs. CR2*)	0.950	0.936–0.960	<0.001
Categorical variables **	**Percent agreement**	**Kappa value (95% CI)**	***p*-value**
*MR1 vs. MR2*			
FHR variability #	99.80%	0.555 (0.208–0.902)	<0.001
FHR acceleration	98.27%	0.848 (0.734–0.959)	<0.001
FHR deceleration	99.75%	0.888 (0.668–1.000)	<0.001
Uterine contraction	98.02%	0.908 (0.846–0.971)	<0.001
*CR1 vs. CR2*			
FHR variability #	99.80%	0.692 (0.433–0.952)	<0.001
FHR acceleration	96.78%	0.862 (0.737–0.989)	<0.001
FHR deceleration	99.50%	0.830 (0.599–1.000)	<0.001
Uterine contraction	99.77%	0.914 (0.858–0.970)	<0.001

* Intraclass correlation coefficient, ** Kappa-Cohen test, # Weighted kappa.

**Table 3:** Agreements in interpretation of fetal heart rate tracings between mobile and conventional fetal cardiotocography.

Continuous Variables *	**ICC**	**95% CI**	***p*-Value**
FHR (*MR vs. CR*, first reviewer group)	0.932	0.916–0.945	<0.001
FHR (*MR vs. CR*, second reviewer group)	0.918	0.900–0.932	<0.001
Categorical variables **	**Percent agreement**	**Kappa value (95% CI)**	***p*-value**
*MR vs. CR* (first reviewer group)			
FHR variability #	99.81%	0.631 (0.292–0.971)	<0.001
FHR acceleration	97.52%	0.808 (0.693–0.924)	<0.001
FHR deceleration	99.50%	0.798 (0.522–1.000)	<0.001
Uterine contraction	87.62%	0.497 (0.378–0.618)	<0.001
*MR vs. CR* (second reviewer group)			
FHR variability #	99.86%	0.800 (0.596–1.000)	<0.001
FHR acceleration	97.03%	0.783 (0.657–0.898)	<0.001
FHR deceleration	99.26%	0.783 (0.521–1.000)	<0.001
Uterine contraction	90.35%	0.581 (0.463–0.699)	<0.001

* Intraclass correlation coefficient, ** Kappa-Cohen test, # Weighted kappa.

**Table 4:** Gwet’s agreement coefficient (AC) assessing agreement for fetal heart rate variability between rater groups and between recording modalities.

	Gwet’s AC	95% Lower	95% Upper	*p*-Value
*MR1 vs. MR2*	0.998	0.995	1.000	<0.001
*MR vs. CR, first reviewer group*	0.998	0.996	1.000	<0.001
*MR vs. CR, second reviewer group*	0.999	0.997	1.000	<0.001
*CR1 CR2*	0.998	0.996	0.999	<0.001

**Table 5:** Quality assessment of fetal heart rate tracings between the two modalities.

Quality	Mobile CTG	Conventional CTG	*p*-Value *
*First reviewer group*			<0.001
Poor (score 0)	21	11	
Acceptable (score 1)	113	90	
Good (score 2)	270	303	
Median score (IQR)	2 (1–2)	2 (1.5–2)	
Mean score ± SD	1.62 ± 0.58	1.72 ± 0.51	
*Second reviewer group*			<0.001
Poor (score 0)	16	5	
Acceptable (score 1)	105	86	
Good (score 2)	283	313	
Median score (IQR)	2 (1–2)	2 (2–2)	
Mean score ± SD	1.66 ± 0.55	1.76 ± 0.45	

* Wilcoxon sign rank test.

**Table 6:** Level of satisfaction assessment of fetal heart rate tracings between mobile and conventional fetal cardiotocography.

Satisfaction Scores	Mobile CTG	Conventional CTG	*p*-Value *
*First reviewer group*			<0.001
1	3	-	
2	19	6	
3	56	31	
4	56	78	
5	268	287	
Median score (IQR)	5 (4–5)	5 (4–5)	
Mean score ± SD	4.41 ± 0.95	4.60 ± 0.70	
*Second reviewer group*			<0.003
1	2	1	
2	7	4	
3	55	31	
4	77	82	
5	263	286	
Median score (IQR)	5 (4–5)	5 (4–5)	
Mean score ± SD	4.46 ± 0.82	4.60 ± 0.69	

* Wilcoxon sign rank test.

## Data Availability

The datasets analyzed during the current study are available from the corresponding author upon reasonable request.

## Abbreviations

The following abbreviations are used in this manuscript:

ACOG	American College of Obstetricians and Gynecologists
BMI	Body mass index
CTG	Cardiotocography
FHR	Fetal heart rate
ICC	Intraclass correlation coefficient
NST	Nonstress test
